# Enhanced photoelectrocatalytic performance of α-Fe_2_O_3_ thin films by surface plasmon resonance of Au nanoparticles coupled with surface passivation by atom layer deposition of Al_2_O_3_

**DOI:** 10.1186/s11671-015-1077-y

**Published:** 2015-09-29

**Authors:** Yuting Liu, Zhen Xu, Min Yin, Haowen Fan, Weijie Cheng, Linfeng Lu, Ye Song, Jing Ma, Xufei Zhu

**Affiliations:** Key Laboratory of Soft Chemistry and Functional Materials of Education Ministry, Nanjing University of Science and Technology, Nanjing, 210094 China; Shanghai Advanced Research Institute, Chinese Academy of Sciences, Shanghai, 201210 China; School of Environmental and Chemical Engineering, Shanghai University, Shanghai, 200444 China

**Keywords:** Hematite, Photoelectrocatalytic water splitting, Surface plasmon resonance, Surface passivation, Atomic layer deposition

## Abstract

The short lifetime of photogenerated charge carriers of hematite (α-Fe_2_O_3_) thin films strongly hindered the PEC performances. Herein, α-Fe_2_O_3_ thin films with surface nanowire were synthesized by electrodeposition and post annealing method for photoelectrocatalytic (PEC) water splitting. The thickness of the α-Fe_2_O_3_ films can be precisely controlled by adjusting the duration of the electrodeposition. The Au nanoparticles (NPs) and Al_2_O_3_ shell by atom layer deposition were further introduced to modify the photoelectrodes. Different constructions were made with different deposition orders of Au and Al_2_O_3_ on Fe_2_O_3_ films. The Fe_2_O_3_-Au-Al_2_O_3_ construction shows the best PEC performance with 1.78 times enhancement by localized surface plasmon resonance (LSPR) of NPs in conjunction with surface passivation of Al_2_O_3_ shells. Numerical simulation was carried out to investigate the promotion mechanisms. The high PEC performance for Fe_2_O_3_-Au-Al_2_O_3_ construction electrode could be attributed to the Al_2_O_3_ intensified LSPR, effective surface passivation by Al_2_O_3_ coating, and the efficient charge transfer due to the Fe_2_O_3_-Au Schottky junctions.

## Background

Solar water splitting has received great attention because of the potential of the mass production of green and renewable fuel [1–3]. The visible-light-active photocatalysts is envisioned to be successful for this application [4]. α-Fe_2_O_3_ (hematite) with low band gap (*E*g = ∼2.2 eV), natural abundance, low cost, and excellent chemical stability is one of the promising metal oxide semiconductor materials for this application [5]. It has been theoretically predicted that a semiconductor with this band gap can achieve a solar-to-hydrogen efficiency of 16.8 % [6]. However, the reported efficiencies of α-Fe_2_O_3_ are notoriously lower than the predicted value, mainly due to the short lifetime of photogenerated charge carriers (<10 ps) [7–9], whereas the absorption depth of 2.2 eV photons (near the band gap) in hematite (118 nm) is much larger than the diffusion distance (2 ~ 4 nm) [10]. In this regard, very thin α-Fe_2_O_3_ films should be used for facilitating the carriers transport and collection. It is significantly crucial and challenging to make the thin film electrode possess efficient absorption for effective photoelectrocatalytic (PEC) water splitting.

Surface plasmon is an efficient method to localize photon absorption at the semiconductor surface through incorporation of plasmonic metal nanoparticles on the semiconductor electrode [11, 12]. Au is an attractive plasmonic metal for PEC water splitting [13], which cannot only interact with the incident light in visible and infrared region but also act as an electron trap facilitating electron–hole separation by forming local Schottky junctions. The plasmon resonance frequency and intensity depends on the geometry and distribution engineering of nanoparticles and also the dielectric property of the surrounding medium.

Surface passivation of semiconductors could efficiently reduce the surface charge recombination in semiconductor technology, which is of significant importance on enhanced performances. The atomic layer deposition (ALD) is a common and easy surface modification method that has been employed in solar cells [14, 15], water splitting [16, 17], and solar fuel production [18, 19]. ALD is a stepwise and conformal coating technique with precisely controlled composition and thickness with a few nanometers. Our recent work demonstrated that 0.8 -fold enhancement of photocurrent was achieved by coating TiO_2_ nanotubes with Al_2_O_3_ [17].

Therefore, it is accordingly hopeful to construct novel structured α-Fe_2_O_3_ electrode with high solar-to-hydrogen efficiency by integrating the surface plasmon resonance and surface passivation on α-Fe_2_O_3_. In this paper, α-Fe_2_O_3_ thin films with surface nanowire were realized by an electrodeposition and post thermally annealing process. Further, Au NPs and Al_2_O_3_ thin layers were loaded on the surface of the α-Fe_2_O_3_ to explore the PEC performance of α-Fe_2_O_3_. Different constructions were achieved with different deposition orders of Au and Al_2_O_3_. Numerical simulations by finite difference time domain (FDTD) method were employed to investigate the effect of Au and Al_2_O_3_ coating on α-Fe_2_O_3_ electrodes.

## Methods

### Synthesis of α-Fe_2_O_3_

Fluorine-modified tin oxide (FTO)-coated glasses were immersed in isopropanol with saturated KOH solution for 24 h to remove absorbed organics, followed by ultrasonically cleaned in acetone, ethanol, and distilled water successively for 25 min. Fe films were prepared by constant current (20 mA • cm^−2^) electrodeposition on the FTO for 120, 180, and 360 s. The deposition solution consists of 48 g ferrous sulfate (FeSO_4_ · 7H_2_O, ≥99.0 %, Greagent), 1.2 g ascorbic acid (C_6_H_8_O_6_, ≥99.7 %, Greagent), 0.4 g amidosulfonic acid (H_2_NSO_3_H, ≥99.0 %, Greagent), 12 g boric acid (H_3_BO_3_, ≥99.5 %, Greagent), and 800 mL distilled water. Electrodeposition was carried out in a standard three-electrode configuration consisting of a Pt foil counter electrode, an Ag/AgCl reference electrode (saturated by 3 M KCl), and a FTO working electrode. After electrodeposition, the α-Fe_2_O_3_ films will be formed by post annealing process in the muffle furnace immediately at 150 °C for 2 h then up to 520 °C for 4 h with a heating rate of 2 °C•min^-1^.

### Loading of Au nanoparticles

The Au films were deposited on the Fe_2_O_3_ electrodes using an ion sputtering equipment (DENTON VACUUM/ DESK V HP) with the current of 30 mA • cm^−2^ for different sputtered time (10, 15, 25, 35 s). The vacuum degree during ion sputtering was lower than 0.099 Torr. The Fe_2_O_3_ electrode modified with nanoparticles (NPs) was denoted as Fe_2_O_3_-Au. Then Fe_2_O_3_-Au electrode was annealed in ambient air at 300 °C for 1 h in a rapid thermal annealing furnace to form Au spheres on the surface of Fe_2_O_3_ [20, 21].

### Conformal coating of Al_2_O_3_

Al_2_O_3_ shells were conformally coated onto the Fe_2_O_3_ or Fe_2_O_3_-Au by ALD processes performed with SUNALETMR-200. Al_2_O_3_ shells were deposited at 200 °C for 25 cycles using Al(CH_3_)_3_ and H_2_O as precursors with a growth rate of ~1 Å•cycle^-1^. Thus, a series of composite nanostructures based on Fe_2_O_3_ films, i.e., Fe_2_O_3_, Fe_2_O_3_-Al_2_O_3_, Fe_2_O_3_-Au, Fe_2_O_3_-Au-Al_2_O_3_, and Fe_2_O_3_-Al_2_O_3_-Au had been constructed.

### Characterization

The morphology and crystalline structure of the electrodes was characterized by field-emission scanning electron microscope (FESEM, Hitachi S4800) and X-ray diffractometer (XRD, Bruker D8 Discover diffractometer), respectively.

The PEC water splitting performances of the Fe_2_O_3_ based electrodes were evaluated by AUTOLAB (PGSTAT302N/FRA2) using a three-electrode setup with the Fe_2_O_3_-based films (1 cm^2^) as working electrode, Ag/AgCl (3 M KCl) electrode as reference electrode, and a platinum foil as counter electrode following our previous work [22]. Used as the supporting electrolyte was 1 M KOH solution. The Al_2_O_3_ layer deposited by ALD method is stable in the KOH solution [23]. The photocurrent was measured at an applied potential of 0.4 V vs Ag/AgCl under chopped light irradiation with a Xe lamp (PLS-SXE300UV) coupled with an AM 1.5G filter. The light was achieved irradiated to the backside of the electrodes (FTO side, backside illumination). The electrochemical impedance spectroscopy measurements were performed in dark at open circuit potential over a frequency ranging from 100000 to 0.1 Hz with amplitude of 10 mV. The Mott-Schottky plots were obtained at a fixed frequency of 1 kHz.

## Results and discussion

**Fig. 1:**
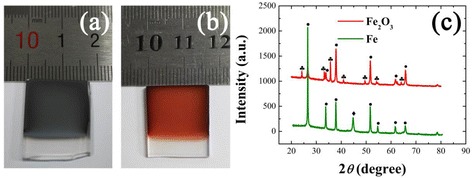
Digital photographs of **a** Fe and **b** α-Fe_2_O_3_ films on the FTO glass; **c** XRD patterns of Fe and α-Fe_2_O_3_ films

Figure 1a, b shows the digital photographs of Fe film with 180 s electrodeposition and Fe_2_O_3_ film after annealing process. The Fe film has a matt black color with good uniformity. Annealing treatment yields the formation of oxide film with red color. Figure 1c shows the XRD patterns of the Fe and Fe_2_O_3_ film. The crystallite diffractions of SnO_2_ from FTO were labeled as solid circle. The XRD pattern of Fe shows the body-centered cubic structure of Fe crystal (JCPDF No. 00-006-0696). The post annealing process converted Fe to hematite structure completely, where the peaks at 35.7°, 33.2°, and 24.2° correspond to the (101), (104), and (012) plane of hematite, respectively (JCPDF No. 33-0664).

**Fig. 2:**
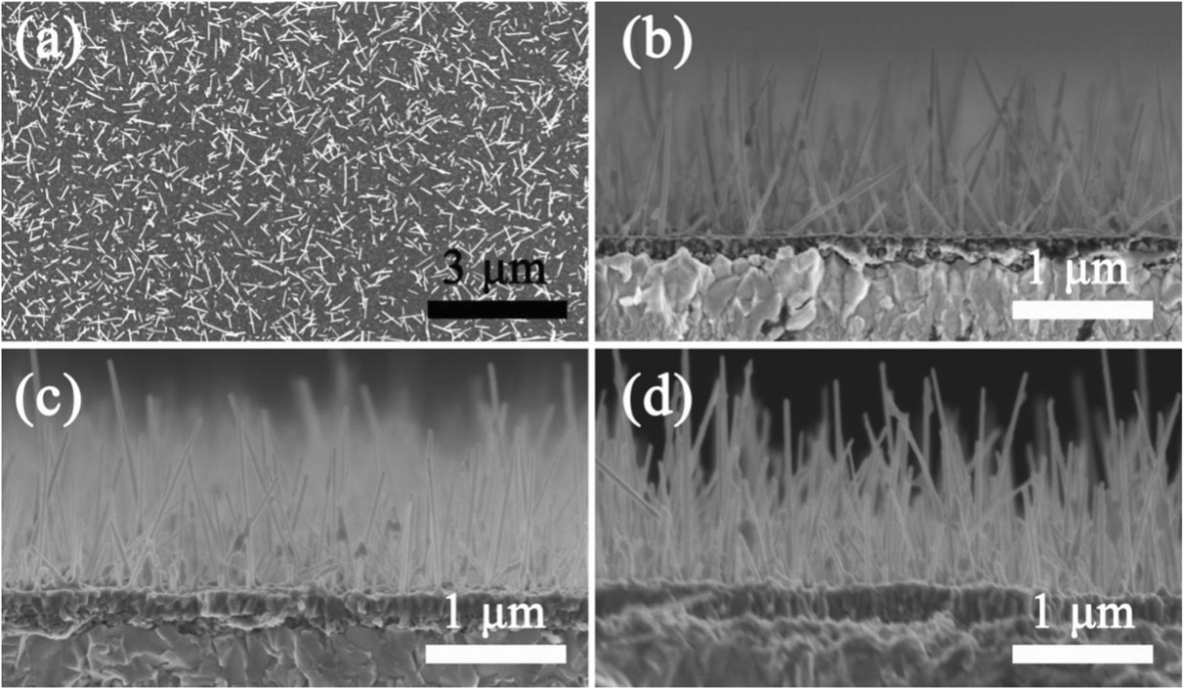
**a** Top view of α-Fe_2_O_3_ with electrodeposition time of 180 s; cross-sectional view of α-Fe_2_O_3_ with different deposition time **b** 120 s; **c** 180 s; **d** 360 s, the electrodeposition current is 20 mA · cm^−2^

Figure 2 shows the top and cross-sectional view of α-Fe_2_O_3_ on the FTO. The thickness of α-Fe_2_O_3_ film was increased from 180 to 270 nm with electrodeposition time increase from 120 to 360 s (Fig. 2b, c). The Fe film formation by electrodeposition is a chemical equilibrium between the chemical dissolution of Fe in the acid deposition solution and the deposition of Fe on the FTO, which is accomplished only as the deposition rate is higher than the surface dissolution rate [24]. The nanowire arrays with diameter of ~40 nm were found to exist uniformly on the surface of oxide thin film. The formation of surface nanowires is ascribed to the vapor-solid oxidation approach on Fe films, which has been reported to be a useful method for vertical growth of α-Fe_2_O_3_ nanowires or nanorods [25].

**Fig. 3:**
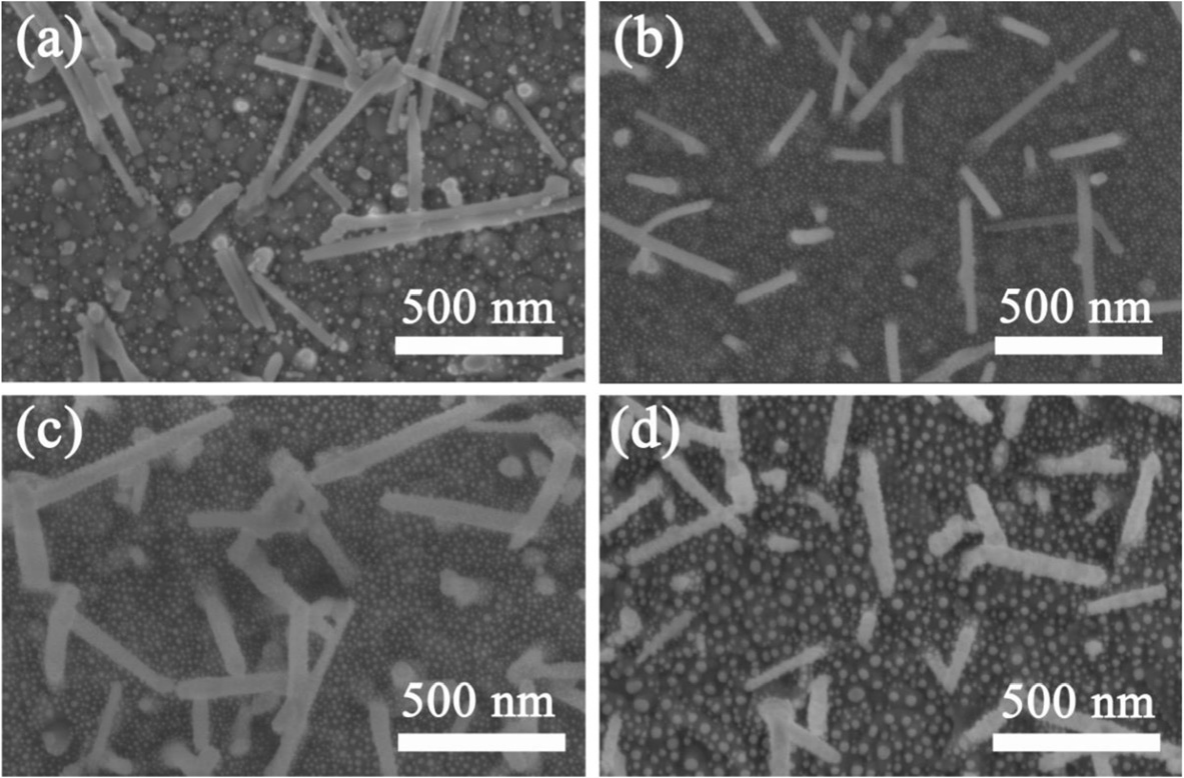
Surface morphology of Fe_2_O_3_-Au electrodes with different Au sputtering time. **a** 10 s; **b** 15 s; **c** 25 s; **d** 35 s. The sputtering current is 30 mA • cm^−2^. All electrodes are annealed at 300 °C for 1 h after the sputtering of Au

Figure 3 shows the surface morphology of Fe_2_O_3_-Au electrodes with different Au sputtering time. It was found that the diameter of Au NPs increases with the sputtering time. The Au NPs sputtered for 10 s are not homogeneous distributed with diameter ranging from 2 to 20 nm (see Fig. 3a). The sample with Au sputtered for 15 s shows the best homogeneous distribution of NPs with diameter of 10 ± 1 nm (see Fig. 3b). Some NPs on the nanowires can also be observed. The Fe_2_O_3_-Au with Au sputtered for 25 s shows the Au diameters with the range of 7–18 nm, while the diameter of sample with Au sputtered for 35 s is up to 40 nm which can be clearly observed in Fig. 3d.

**Fig. 4:**
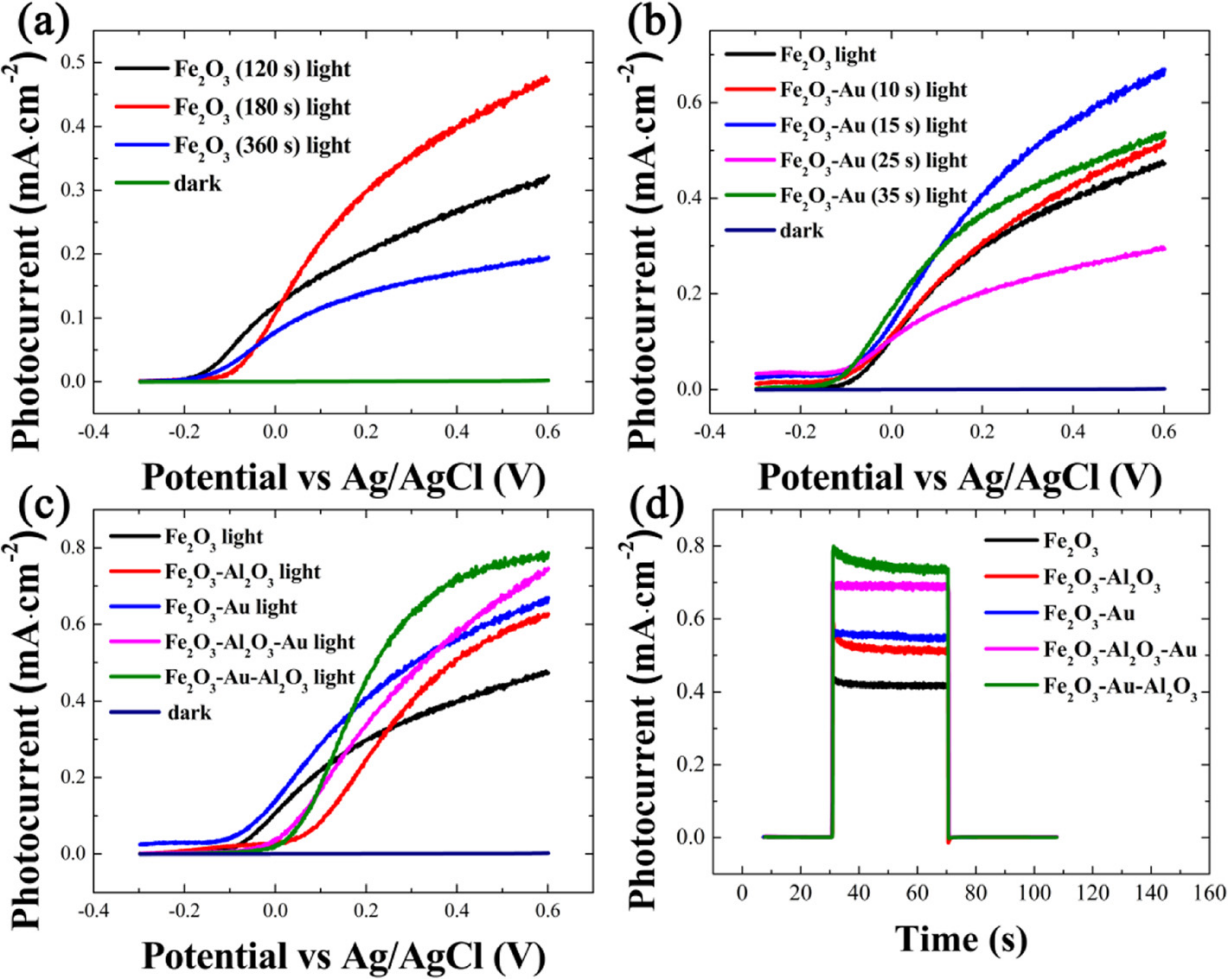
LSV curves of **a** pristine Fe_2_O_3_ electrode with varying deposition time, **b** pristine Fe_2_O_3_ and Fe_2_O_3_-Au electrodes with different Au sputtering time, and **c** pristine Fe_2_O_3_ and modified Fe_2_O_3_. **d** Photocurrent responses of pristine Fe_2_O_3_ and modified Fe_2_O_3_ at an applied potential of 0.4 V. Electrolyte, 1 M KOH solution, illumination: a 300 W Xe lamp coupled with an AM 1.5 filter

The linear sweeps voltammetry (LSV) curves of the pristine Fe_2_O_3_ electrodes with varying deposition durations are shown in Fig. 4a. The photocurrent of the Fe_2_O_3_ electrode with deposition time for 180 s is the highest of all above −0.15 V vs Ag/AgCl. When the deposition time is 120 s, the thickness of film is ~180 nm (seen in Fig. 2b) which is too thin to absorb sufficient light. As the deposition time increases to 360 s, the photocurrent is even lower, as seen from the Fig. 4a. The short hole diffusion length (∼2–4 nm) [9] allows only holes created close to the electrolyte interface to oxidize water. Since the light penetration length in α-Fe_2_O_3_ is of the order of 100 nm [26], most holes created in the bulk will recombine with electrons before reaching the surface as the thickness of Fe_2_O_3_ film increases to 270 nm (see Fig. 3d). In this regard, the following α-Fe_2_O_3_ photoelectrodes are studied based on 180 s deposition unless otherwise stated.

The LSV curves of the α-Fe_2_O_3_ electrodes with different Au sputtering durations are shown in Fig. 4b. The Fe_2_O_3_-Au (15 s) shows the best PEC performance among these electrodes above 0.1 V vs Ag/AgCl. It may be resulted from the particle size and the distribution variations for the different Fe_2_O_3_-Au samples, which changes the resonance frequency and intensity of Au SPR peaks [27]. As the nanoparticles are smaller than 3 nm, the SPR could not be motivated due to the quantum confinement effect [28]. The SPR begins to work as the nanoparticles increase to be larger than 4 nm, and the resonance will be red shifted with the size increase of metal nanoparticles. The spectra overlap between plasmonic Au nanoparticles, and Fe_2_O_3_ may happen on the proper Au nanoparticles morphology. Additionally, the metal nanoparticles absorb photons from an area much larger than their geometric cross section [29]. The photon absorption by Fe_2_O_3_ itself may be decreased with the excessive size increase of metal particles. According to the experimental results, 15 s was thus chosen as the sputtering time for the following Au deposition.

Le Formal et al*.* [23] demonstrated an enhancement of photocurrent in comparison with the Fe_2_O_3_ electrode by employing Al_2_O_3_ passivation coating. The Al_2_O_3_ surrounding also results in an increase of dielectric circumstance of metal nanoparticles, which could strengthen the localized electromagnetic field with tunable resonance frequency [30, 31]. Therefore, a combination of NPs and Al_2_O_3_ coating is of special interest in PEC system. For comparison, the modified Fe_2_O_3_ electrodes with both single step coating (i.e., Fe_2_O_3_-Al_2_O_3_ and Fe_2_O_3_-Au) and sequential coating (Fe_2_O_3_-Au-Al_2_O_3_ and Fe_2_O_3_-Al_2_O_3_-Au) processes are characterized. Figure 4c shows that the photocurrent of the pristine Fe_2_O_3_-Au electrodes is obviously higher than others at 0.0 V vs Ag/AgCl, which could be attributed to the increased open circuit potential by the Au modification. The hematite films are n-type photoanodes and a positive applied bias potential will increase the photocurrent generation as the Fermi level moves to assist charge separation and facilitate water splitting [32]. The conduction band of α-Fe_2_O_3_ is located slightly below the level needed for hydrogen production, and its valence band is well-suited for oxygen production. Therefore, an external bias is typically required for water splitting when using α-Fe_2_O_3_ materials as photoanodes [26, 33]. The photocurrent of the samples increases unceasingly at a higher potential, and the photocurrent of the Fe_2_O_3_-Au-Al_2_O_3_ electrode increases to 1.78-fold compared to that of the pristine Fe_2_O_3_ at the potential of 0.4 V. The trend of photocurrent responses measured at an applied potential of 0.4 V in Fig. 4d mirrors that of the LSV plots, which follows the order of Fe_2_O_3_-Au-Al_2_O_3_ > Fe_2_O_3_-Al_2_O_3_-Au > Fe_2_O_3_-Au > Fe_2_O_3_-Al_2_O_3_ > Fe_2_O_3_.

**Fig. 5:**
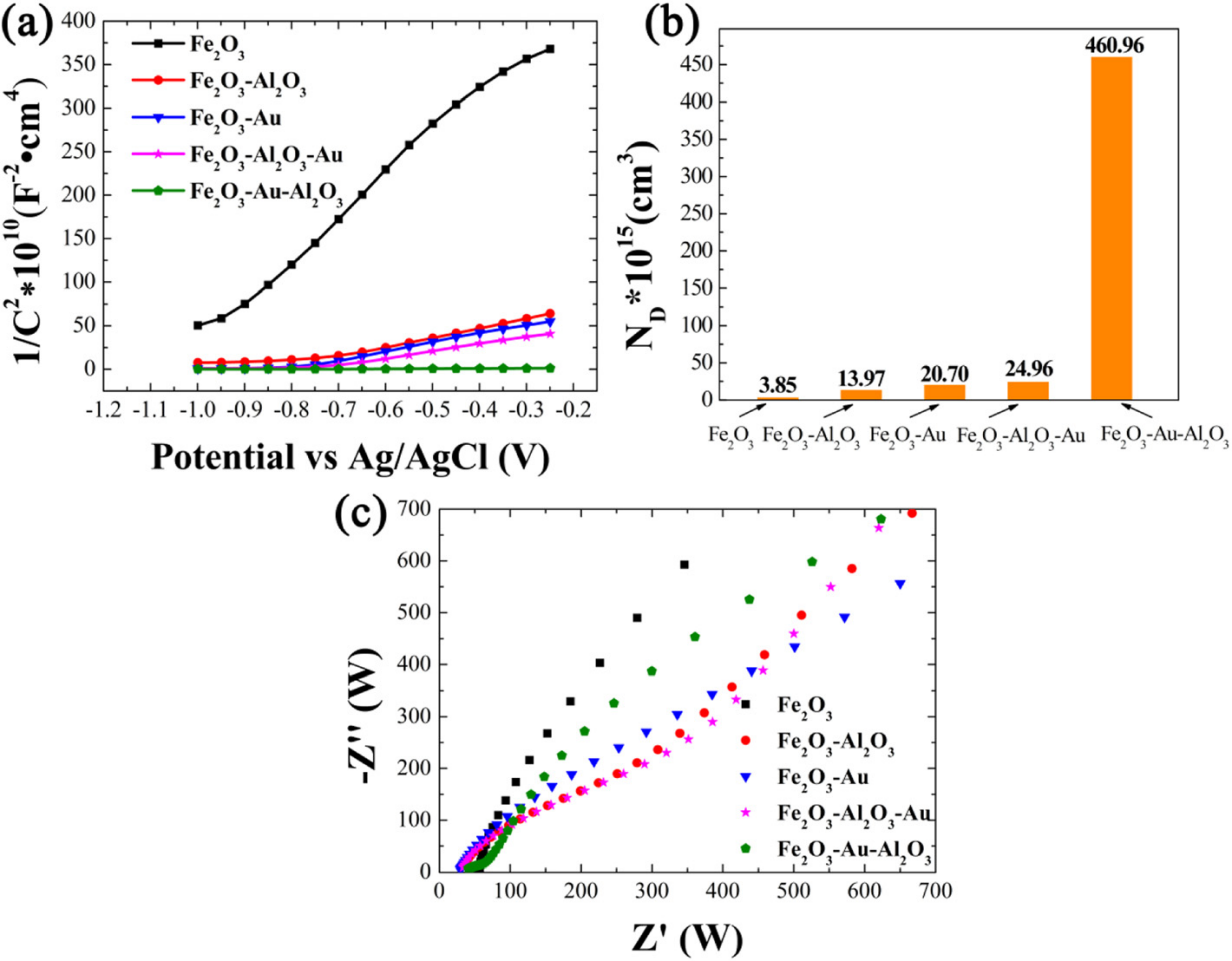
**a** Mott–Schottky plots of five different electrodes at a fixed frequency of 1 kHz in 1 M KOH solution under dark condition; **b** charge carrier density (N_D_) obtained from Mott-Schottky analysis, **c** Nyquist plots of five electrodes in 1 M KOH solution under dark condition

The Mott-Schottky and Nyquist plots are used to determine the carrier density, capacitance, and impedance of the electrodes, and the results are shown in Fig. 5. Carrier density can be calculated from the slope of Mott-Schottky plots. The equation is shown as follows:

$$ {N}_{\mathrm{d}}=\frac{2}{e_0\varepsilon {\varepsilon}_0\frac{\mathrm{d}\left(\frac{1}{C^2}\right)}{\mathrm{d}V}} $$

Where *e*_0_ is the electron charge, *ε* is the dielectric constant of Fe_2_O_3_ (*ε* = 80) [34, 35], *ε*_0_ is the permittivity of vacuum, *N*_d_ is the donor density, and *V* is the applied bias at the electrode. The positive slopes indicate the n-type behaviors of both pristine and modified samples. The calculated electron density of electrodes is shown in the Fig. 5b. The electron density of Fe_2_O_3_-Au-Al_2_O_3_ is 4.61 × 10 cm^−3^ [17], which is 120 times of that of pristine Fe_2_O_3_ electrode. Then, the interfacial properties between the electrolyte and electrodes are further characterized by electrochemical impedance spectroscopy (EIS) under dark condition (Fig. 5c). The arc of Nyquist plot is characteristic of charge transportation resistance. The diameter of the arc for the Fe_2_O_3_-Au-Al_2_O_3_ electrode is the smallest one, indicating that the resistance of the charge transportation is significantly decreased. The diameter of the arc for these electrodes follows the order of Fe_2_O_3_-Au-Al_2_O_3_ < Fe_2_O_3_-Al_2_O_3_-Au ≈ Fe_2_O_3_-Al_2_O_3_ < Fe_2_O_3_-Au < Fe_2_O_3_. The small arc diameter of Al_2_O_3_ coated Fe_2_O_3_-based electrode indicates the facilitated charge transportation enhanced by the decreased surface recombination from Al_2_O_3_ passivation.

**Fig. 6:**
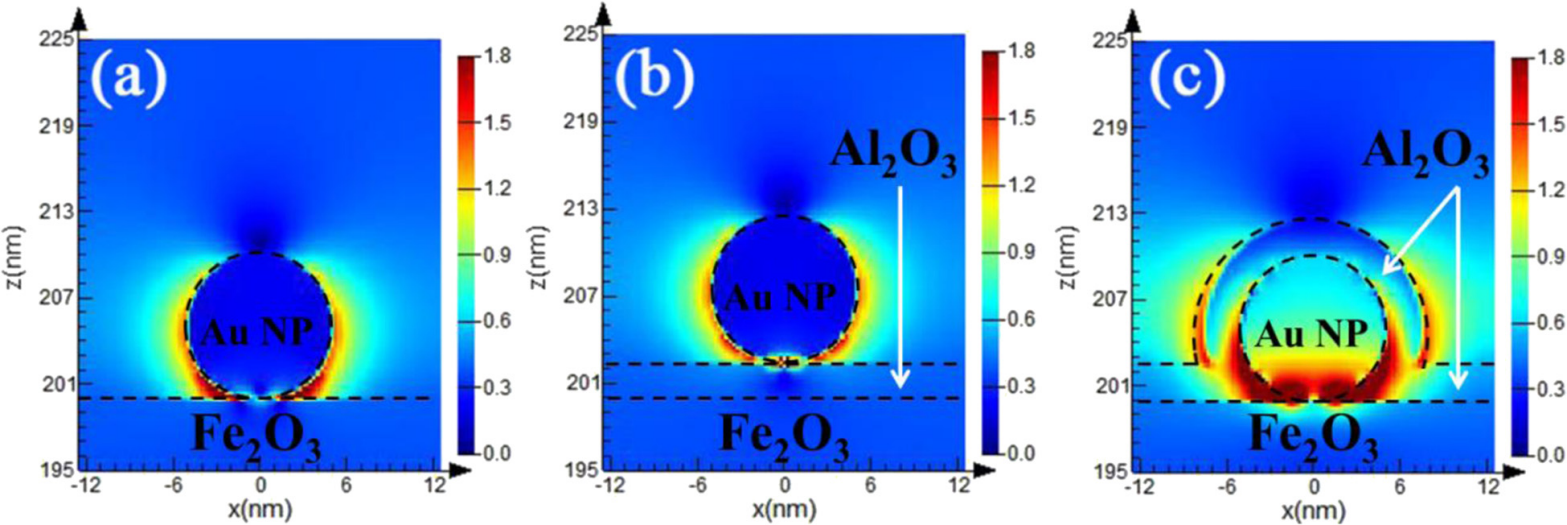
Cross-sectional electric field intensity distributions for five electrodes at an incident light wavelength of 574 nm, **a** Fe_2_O_3_-Au, **b** Fe_2_O_3_-Al_2_O_3_-Au, and **c** Fe_2_O_3_-Au-Al_2_O_3_. The size of Au NP is 10 nm

The FDTD simulations were performed to calculate electric field distribution across the interfaces in different electrodes under 574 nm as shown in Fig. 6. The color index represents the magnitude of electric field intensity normalized with that of the light propagating in free space. The electric field intensity of pristine Fe_2_O_3_ and Fe_2_O_3_-Al_2_O_3_ electrodes is very weak under 574 nm, and there is no change in color on the interface (not shown here). As coupling the Au plasmonic structure and Al_2_O_3_ coating on Fe_2_O_3_ electrode, high-electric field intensity can be always found in the area where the Au is in contact with semiconductor and Al_2_O_3_. It is generally accepted that there are three non-mutually exclusive energy-transfer mechanisms in plasmonic-photocatalyst systems, involving the SPR-induced charge injection from metal to semiconductor, near-field electromagnetic, and scattering mechanism [29]. The scattering effect can be safely ruled out in our study because it usually occurs in plasmonic metal nanostructures with the diameter larger than 50 nm. SPR-induced charge injection and near-field electromagnetic mechanism may co-contribute on our Fe_2_O_3_-Au systems. In near-field electromagnetic mechanism, the excited Au increases the intensity of local electric field, which will penetrate into Fe_2_O_3_ and amplify the local light intensity. For the Fe_2_O_3_-Al_2_O_3_-Au electrode, the intensified electromagnetic field is largely blocked by the Al_2_O_3_ spacer layer. This suggests that the near-field electromagnetic enhancement mechanism can hardly contribute the enhanced PEC performance with the presence of dielectric spacer layer, which is in accordance with our previous work [16]. Despite this, the Fe_2_O_3_-Al_2_O_3_-Au electrode still shows the higher photocurrent than Fe_2_O_3_-Al_2_O_3_ electrode, which could benefit from the SPR-mediated hot-electron injection process. The Fe_2_O_3_-Au-Al_2_O_3_ electrode shows the strongest LSPR electric field, biggest radiation areas, and deepest penetration depth into the Fe_2_O_3_ (Fig. 6c)_,_ as compared with the other configurations. This may be mainly resulted from the fact that the Al_2_O_3_ coating increases the refractive index of the surrounding medium from 1.33 (in water) to 1.76 (in Al_2_O_3_) [36, 37], which could intensify the plasmon resonance. Both near-field electromagnetic effect and SPR-induced charge injection from metal to semiconductor could contribute the intensified electric field and the enhanced PEC performances. The rate of electron–hole formation is proportional to the local intensity of the electric field and radiation areas [38]. It means that more pairs of electron–hole are generated in the Fe_2_O_3_ semiconductor, which is in accordance with the much increased ND value in the Mott-Schottky analysis (see Fig. 5). Consequently, most of the photogenerated charges created by the plasmon excitation contribute to the surface catalysis for water splitting. From the perspective of the SPR-mediated hot-electron injection mechanism, the intensified electromagnetic field will facilitate more energetic electrons on Au nanoparticles. Electrons photoexcited by the Au NPs will pass over the Schottky barrier and migrate to the conduction band of Fe_2_O_3_. Schottky barrier at the interface also helps the transferred hot electrons accumulate in the Fe_2_O_3_ conduction band, preventing them from traveling back to the Au NPs.

## Conclusions

The thin film α-Fe_2_O_3_ electrodes with surface nanowire were successfully obtained by electrodeposition and post thermal annealing process for PEC water-splitting application. The LSPR of Au NPs in conjunction with surface passivation by Al_2_O_3_ shells was further introduced for the enhanced PEC performance of α-Fe_2_O_3_ photoelectrodes. Among the different configurations, the Fe_2_O_3_-Au-Al_2_O_3_ construction shows the best PEC performance, attributing to the Al_2_O_3_ intensified LSPR, effective surface passivation by Al_2_O_3_ surface coating, and the rapid charge carriers transfer due to the Schottky junctions at the interface of metal and semiconductor. These results can not only contribute fundamentally to the mechanism studies of the SPR-based photocatalysis but also open a new avenue for the design strategies of high-performance photocatalysts for solar-to-fuel energy conversion.
